# Comparative Analyses of Different Routes to Prepare Cutin Colloidal- and Nano- Particle Dispersions from Tomato (*Solanum lycopersicum*) Peels

**DOI:** 10.3390/polym17172348

**Published:** 2025-08-29

**Authors:** Sandra Bučko, Ljiljana Spasojević, Jelena Milinković Budinčić, Jadranka Fraj, Lidija Petrović, Jaroslav Katona, Saule Aidarova, Kuanyshbek Mussabekov, Alpamys Babayev, Raziya Sarsembekova, Altynay Sharipova

**Affiliations:** 1 Faculty of Technology Novi Sad, University of Novi Sad, Bul. cara Lazara 1, 21000 Novi Sad, Serbia; lj.spasojevic@tf.uns.ac.rs (L.S.); jelenamilinkovic86@yahoo.com (J.M.B.); jadrankam@gmail.com (J.F.); lidijap@uns.ac.rs (L.P.); jaroslav.katona@uns.ac.rs (J.K.); 2 Petroleum Engineering Institute “One Belt, One Road”, Kazakh–British Technical University, Tole bi str. 59, Almaty 050000, Kazakhstan; aidarova@mail.ru (S.A.); musabekov40@mail.ru (K.M.); a_babayev@mail.ru (A.B.); r.sarsembekova@kbtu.kz (R.S.); 3 Mining and Metallurgical Institute, Satbayev University, Satbayev str. 22a, Almaty 050013, Kazakhstan; a_sharipova85@mail.ru

**Keywords:** cutin nanoparticles, colloid particles, natural polyester, nanoparticle stability, *Solanum lycopersicum*, cutin dispersion

## Abstract

Cutin is a natural plant polyester, a constituent of the cuticle that covers aerial plant surfaces. Following the trends of agricultural and food waste reduction and the growing demand for plant-derived nanomaterials, cutin was extracted from tomato peels, a by-product of tomato processing. Subsequently, dispersions of cutin particles in the nano- and colloidal size range were prepared by pH-dependent precipitation. Four types of the dispersions were obtained, i.e., dispersion from cutin extract—NP E dispersion, dispersions from a solution of different cutin isolates, dialyzed cutin isolate–NP D dispersion, washed cutin isolate–NP W dispersion, and standard cutin isolate–NP S dispersion. Cutin precipitation occurred at *pH* lower than 7 and cutin dispersions with final *pH* 3–7 were formed. Zeta potential, particle size, and recovery of four cutin dispersions were investigated. All types of cutin particles bear a negative charge which increases on *pH* increase from 3 to 7, resulting in decrease in cutin nanoparticle size upon *pH* increase. In addition to that, the influence of cutin solution concentration and storage time on cutin dispersion particle size was found to be mitigated at *pH* ≥ 6. Among four dispersions, NP S had the highest cutin nanoparticle recovery at all *pH*s investigated.

## 1. Introduction

Nature remains a central focus of scientific and technological research, especially in nanotechnology, which has attracted significant attention in recent years due to its remarkable properties [1]. Nanoparticles can be used to encapsulate and protect bioactive ingredients, to modify stability, texture and optical properties of food products, to control the release of nutrients within the gastrointestinal tract, act as a sensor for microbiological spoilage detection in food products, stabilize emulsions and Pickering emulsions, and to form edible films [2,3,4]. There are a number of techniques used in nanoparticle fabrication, but they are all categorized into two main groups, the top-down approach and the bottom-up approach, according to the principle lying behind the technique [5]. Top-down approach techniques involve breaking down the bulk materials or larger particles into nanoparticles by application of disruptive forces such as shear, impact, and compression [6,7]. On the contrary, within bottom-up approach techniques, nanoparticles are built by assembling molecules or smaller particles due to changes in environmental conditions, such as pH, ionic strength, temperature, and solvent concentration. The advantages of bottom-up approach techniques in comparison to top-down approach techniques are lower energy input requirements, lower cost, improved control over particle properties, and significantly reduced risk of sample contamination [5,6,8,9]. Such principles go along with the concept of green technology and methods that are environmentally responsible, economical, and use sustainable strategies to reduce waste production [10].

Depending upon the nature of their building material, nanoparticles can be grouped into various categories, including inorganic nanoparticles, organic nanoparticles, and combined organic/inorganic or surface-modified nanoparticles. However, recently there has been an emerging interest in the development of biomaterial-based nanoparticles. Studies have shown that a variety of organic and edible nanoparticles can be prepared from food-based ingredients, such as polysaccharides, lipids, proteins, minerals, and surfactants [4,11]. The important area of current research is the identification of new sources of biopolymers that are compatible with food and beverage matrices and suitable for large-scale nanoparticle production [4,12].

One of such biopolymers is cutin, the most abundant lipid polyester in vascular plants, constituent of the cuticle that covers aerial plant surfaces [13,14,15]. Cutin is composed of glycerol and C16–C18 interesterified fatty acid derivatives, such as hydroxy and hydroxyl-epoxy substituted fatty acids [15,16,17,18]. A suite of physical, chemical, and morphological properties gives the plant cutin characteristics of a unique and complex biopolymer [19]. Cutin is a non-toxic, biodegradable, waterproof, UV-blocking, amorphous, insoluble, infusible, and highly available bio-polyester [14,17,20,21,22]. So far, cutin has been isolated from various sources, including plant leaves, fruit peels, and fruits (various varieties of apple, apple pomace, papaya, lime, tomato, green pepper, cucumber, watermelon, different berries, etc.) [23,24,25]. A few years ago, a simple cutin isolation method was proposed—alkaline extraction and subsequent pH-dependent precipitation of cutin from tomato peels that enables one to obtain high-purity cutin products under mild conditions and without the use of organic solvents [24]. Nevertheless, the isolated cutin has been mostly used as an inducer for cutinase production [23,26] or, in combination with other polymers, for preparation of plastic-like materials [14,18,21], but so far, there has been no literature data on bottom-up cutin nanoparticle preparation and only one study on top-down cutin nanoparticle formation [17].

Therefore, in this study cutin was isolated following some principles of green technologies by using agricultural waste—tomato peels—and the bottom-up principle for cutin nanoparticle formation. First, cutin was extracted by alkaline extraction from tomato peels and subsequently cutin nanodispersions were formed by pH-dependent precipitation. Cutin particle dispersions were formed either directly after the extraction during the precipitation step or by the precipitation of the dissolved cutin after being isolated and dried. Different routes of cutin nano and colloidal particle dispersion preparation were analyzed in terms of the influence of *pH* on particle size, zeta potential, and particle recovery. In addition to that, cutin dispersion stability and the influence of cutin concentration on particle size were investigated at different final *pH* (5, 6, and 7).

## 2. Materials and Methods

### 2.1. Materials

Tomato (*Solanum lycopersicum*) peels were obtained from local Serbian farmers. Sodium hydroxide, NaOH (purity ≥ 99%), was obtained from Centrohem, Stara Pazova, Serbia and 36% hydrochloride acid, HCl, was obtained from ZorkaPharm, Šabac, Serbia. Regenerated cellulose membrane was obtained from Demineralized water was used as a solvent.

### 2.2. Preparation of the Cutin Isolate

**Figure 1:**
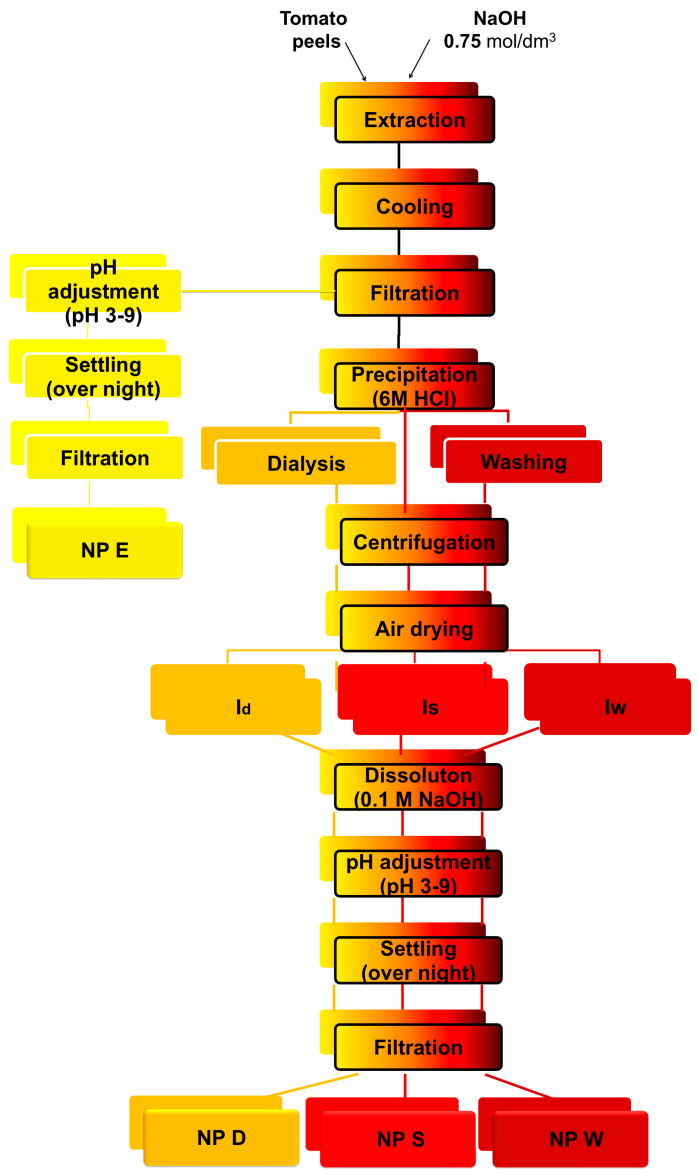
Schematic illustration of different routes to prepare cutin nanoparticle dispersions from tomato (*Solanum lycopersicum*) peels. Nanoparticle dispersion from cutin extract NP E—yellow color, nanoparticle dispersion from dialysed cutin isolate NP D—orange, nanoparticle dispersion precipitated from wahed cutin isolate NP W—dark red, and standard nanoparticle dispersion NP S—red.

Tomato peels were washed from tomato seeds and juice leftovers using tap water and consequently air dried at room temperature. Cutin extraction was carried out by employing the procedure of Cigognini et al. [27]. Cutin was extracted from dried tomato peels by alkaline extraction. Firstly, tomato peels were suspended in 0.75 mol/dm^3^ NaOH solution (mass ratio tomato peels: NaOH = 1:19) and vigorously stirred with a mechanical stirrer at a temperature of 90 °C for two hours to allow cutin dissolution. The obtained dispersion of tomato peels was then cooled down to room temperature in a water bath and filtered through quantitative filter paper to separate remains of tomato peels and obtain cutin extract. Cutin was precipitated from the extract by the addition of the required volume of 6 mol/dm^3^ HCl to cutin extract to precipitate cutin at *pH* 5 and obtain precipitated cutin dispersion, which was then subjected to one of three steps (Figure 1):

1. Dialysis of the precipitated cutin dispersion through dialysis tubing cellulose membrane, D9402–100FT, Sigma-Aldrich, St. Louis, MO, USA. Conductivity of the precipitated cutin dispersion was monitored and dialysis was stopped when conductivity had dropped down to ≤15 μS. Conductivity was measured on Cond Level 1 Conductometer, InoLab, Weilheim, Germany.
2. Washing—precipitated cutin was washed in demineralized water (mass ratio cutin precipitate:water = 1:100) by gentle stirring for 20 min to obtain cutin dispersion which was subsequently centrifuged at 4 °C and 10,000 rpm for 20 min to separate cutin from liquid. Washing/centrifugation steps were repeated three times. Centrifugation was carried out using a Sigma 4–16KS centrifuge (Osterode am Harz, Germany).
3. No action taken; the precipitated cutin dispersion was subjected to the next step.

The precipitated cutin dispersions obtained after dialysis, washing, or directly after the precipitation step were subjected to centrifugation at 4 °C and 10,000 rpm for 20 min to separate precipitated cutin from the liquid phase. Precipitated cutin was then spread into a thin layer and let dry at room temperature to obtain dialyzed cutin isolate—I_d_, washed cutin isolate—I_w_, and standard cutin isolate—I_s_, depending on the step that the cutin dispersion was subjected to.

### 2.3. Cutin Extract Yield

Cutin extract yield is mass of cutin present in the extract that was obtained from 100 g of tomato peels, expressed as a mass percentage. It was calculated by the following Equation (1):

*Yield* (%) = (c*_CE_*·*m_CE_*)·100/*m_TP_* (1)

where *m_CE_* is the mass of cutin extract obtained from 100 g of tomato peels (*m_TP_*), while *c_CE_* presents cutin concentration in the extract. Cutin concentration in the extract was calculated according to Equation (2):

*c_CE_* (%) = ((*m_DCE_*/*m_CE_*) − (*m_DS_*/*m_S_*)) · 100 (2)

where *m_DCE_* is the mass of cutin extract dry matter present in *m_CE_* taken for the analysis, while *m_DS_* stands for the mass of solvent (NaOH solution that was used for the cutin extraction) dry matter present in *m_S_*—mass of solvent taken for the analysis. Dry matter was determined according to the AOAC Standards [28]. The reported values are the mean values of at least three measurements.

### 2.4. Cutin Isolates Yield

Cutin isolate yield presents mass of cutin isolate obtained from 100 g of tomato peels, expressed as a mass percentage. It was calculated according to Equation (3):

*Yield* (%) = *m_CI_*·100/*m_TP_* (3)

where *m_CI_* presents mass of cutin isolate obtained from *m_TP_*—mass of tomato peels.

### 2.5. Cutin Isolate Ash and Moisture Content

Cutin isolate ash and moisture content were determined according to the AOAC Standards [28]. The reported values are mean value of at least three measurements.

### 2.6. Preparation of the Cutin Particle Dispersions

Cutin particle dispersions were prepared either by cutin precipitation directly from the cutin extract—nanoparticle dispersion NP E, or by the cutin precipitation from the cutin isolate solution: nanoparticle dispersion NP D precipitated from solution of dialyzed cutin isolate, I_d_, nanoparticle dispersion NP W precipitated from solution of washed cutin islate, I_w_ and nanoparticle dispersion NP S precipitated from of standard cutin isolate, I_s_, solution Figure 1.

NP E were prepared by the addition of the required volumes of 6 mol/dm^3^ HCl to the cutin extract (*pH* = 14) of the required concentration (1–2.85%, which was adjusted by the addition of the 0.75 mol/dm^3^ NaOH) in order to bring about controlled precipitation of the cutin and obtain NP E at *pH* 3–9.

For preparation of NP D, NP W, and NP S, the required mass of I_d_, I_w_, or I_s_, respectively, was suspended in 0.1 mol/dm^3^ NaOH solution (*pH* = 13) and stirred for 2 h at room temperature to allow cutin isolate dissolution and to obtain cutin isolate solutions of different concentrations, *c_s_* from 1% to 4%. The *pH* of cutin isolate solutions was carefully adjusted to *pH* 3–9 by 1 mol/dm^3^ HCl to bring about controlled precipitation of the cutin and thereby to obtain dispersions of cutin nanoparticles.

Cutin particle dispersions were left overnight to settle and were filtered through quantitative filter paper afterwards, to remove larger particles while the filtrates were used in further experiments.

### 2.7. Cutin Particles Recovery

Cutin particle recovery, *R*, presents a mass percentage of cutin extract or cutin isolate recovered in the form of particles within cutin particle dispersions. *R* was calculated by Equation (4):

*R* (%) = (*c_NP_*/*c_s_*) · 100 (4)

where *c_NP_* is the concentration of the cutin particles in the dispersion and *c_s_* presents the concentration of cutin isolate solution (in case of NP E, it is equal to cutin concentration in the extract, *c_s_* = *c_CE_*).

Cutin particle concentration was calculated as Equation (5):

*c_NP_* (%) = ((*m_DNP_*/*m_ND_*) − (*m_DS_*/*m_S_*)) · 100 (5)

where *m_DNP_* is the mass of particle dispersion dry matter present in mass of particle dispersion taken for the analysis, *m_ND_*. The reported values are the mean values of at least three measurements.

### 2.8. Particle Size and Zeta Potential Measurements

Zeta potential (*ζ*) and mean particle diameter (*d*) of NP I, NP S, NP D, and NP W at different *pH* were determined using Zetasizer Nano ZS (Malvern Instruments, Worcestershire, UK). Folded capillary cell (DTS 1060) was used for zeta potential measurements, and disposable polystyrene cuvette (DTS 0012) for the particles’ size measurements. Cutin particle dispersions of different *pH* (3–9) were diluted to 1:50 by a corresponding dilution matrix. The dilution matrix was obtained by the addition of NaOH solution (*c_NaOH_* = 0.75 mol/dm^3^ for NP E and *c_NaOH_* = 0.1 mol/dm^3^ for NP D, NP S, and NP W) of corresponding *pH*, where *pH* was adjusted by the addition of HCl (*c_HCl_* = 6 mol/dm^3^ for NP E and *c_HCl_* = 1 mol/dm^3^ for NP S, NP D, and NP W). The *pH* of the diluted nanoparticle dispersions was checked and, if needed, readjusted to match the nominal value. All measurements were carried out in triplicate.

### 2.9. Cutin Particle Dispersion Stability

Cutin particle dispersion stability was evaluated by measuring particle size of NP E, NP S, NP D, and NP W (*c_s_* = 2%) of different *pH* (5, 6, and 7) during 14 days of the nanoparticle dispersion storage at room temperature. All measurements were carried out in triplicate.

## 3. Results and Discussion

Cutin nanoparticle dispersions were prepared by the cutin precipitation from the cutin extract—nanoparticle dispersion NP E, or by the cutin precipitation from the solution of one of three cutin isolates, nanoparticle dispersion NP D, NP W, and NP S from the dialyzed (I_d_), washed (I_w_), and standard (I_s_) cutin isolate, respectively (Figure 1).

As Figure 1 shows, preparation of the NP E is the simplest and the fastest way to obtain cutin nanoparticles. The characteristic of NP E is their high ionic strength and relatively low cutin particle concentration, which both depend on the cutin extraction conditions, e.g., on the concentration of the NaOH solution. On the other hand, preparation of the cutin dispersion from the cutin isolates allows cutin precipitation from the solution of higher cutin concentration than in cutin extract. During preparation of the I_w_ and I_d_ cutin isolates, the additional steps, washing and dialysis, respectively, were taken to further reduce the ionic strength of the final nanoparticle dispersion. It turned out that dialysis is the most efficient way to reduce the ionic strength (results presented below); however, it is a very time-consuming process.

### 3.1. Cutin Isolates’ Yield, Ash, and Moisture Content

**Figure 2:**
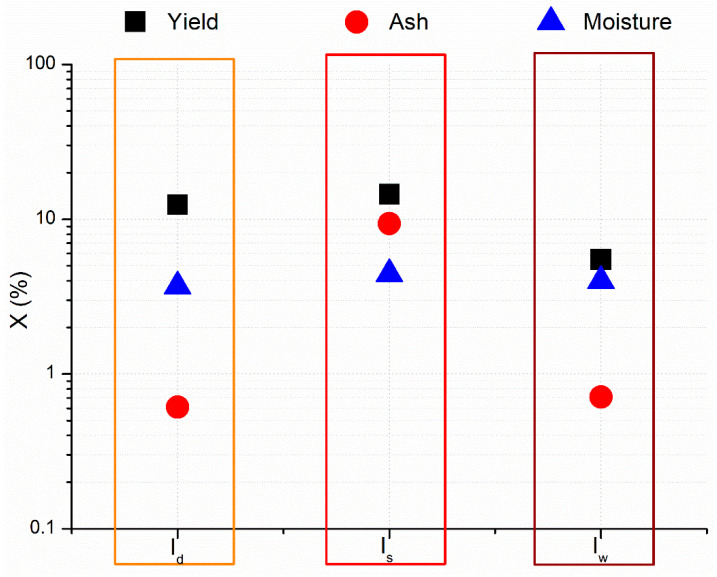
Yield (black square), ash (red circle), and moisture content (blue triangle) of I_d_, I_s_, and I_w_ cutin isolates.

Characteristics of the cutin isolates in terms of yield, ash, and moisture content and the effects that the additional steps, such as washing and dialysis, have on these properties are shown in Figure 2.

Yield of the cutin isolates follows the descending order: I_s_, I_d_, and I_w_ cutin isolate. The yield of I_s_ cutin isolate of 14.5% was only slightly higher than the yield of I_d_ cutin isolate of 12.4%, but significantly higher than the yield of I_w_ cutin isolate of 5.5%. The lowest yield of I_w_ cutin isolate was attributed to a gradual loss in cutin during the three successive washings. The theoretical maximal yield that could be obtained, i.e., the yield of cutin in the cutin extract used for cutin precipitation, was 28.5%. Yield of I_s_ and I_d_ cutin isolate corresponds to the values reported by [27] of around 15% for cutin isolate from tomato peels, but it is significantly lower than the yield of 25 ± 2% obtained by Manrich et al. (2017) [17].

Ash content of cutin isolates was 0.61%, 0.71%, and 9.39% for I_d_, I_w_, and I_s_, respectively. The significantly lower ash content present in I_d_ and I_w_ in comparison to I_s_ cutin isolate is a result of dialysis and washing, which were used with the aim to reduce concentration of acid/alkaline present within cutin isolate.

The moisture content of I_d_, I_w_, and I_s_ cutin isolates was found to be 3.68 ± 0.18%, 3.99 ± 0.20%, and 4.43 ± 0.20%, respectively.

### 3.2. Colloidal Properties of Cutin Particle Dispersions

**Figure 3:**
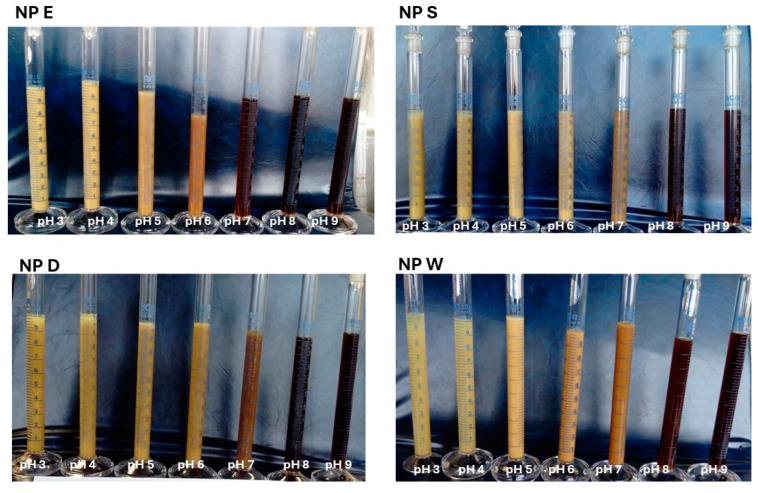
Influence of *pH* (3–9) on the appearance of cutin particle dispersions NP E, NP S, NP D, and NP W. *c_S_* = 2%.

Different preparation routes, cutin precipitation from the cutin extract or from the cutin isolate solution, were employed to obtain dispersions of cutin nanoparticles where cutin nanoparticles were formed by controlled precipitation of dissolved cutin upon a decrease in cutin solution’s *pH* from *pH* = 13 to a targeted *pH* (3–9). The influence of *pH* (3–9) on the appearance of thus obtained nanoparticle dispersions, NP E, NP S, NP D, and NP W, is presented in Figure 3.

As can be seen in Figure 3, upon *pH* decrease from 9 to 3, cutin solution appearance turned from a dark brown, clear cutin solution (*pH* ≥ 8) to a light yellowish, turbid cutin dispersion (*pH* ≤ 7). Namely, *pH* decrease resulted in cutin precipitation, which is presented by the lighter color, at *pH* ≤ 7 apart from NP E, where precipitation started at *pH* = 6. Therefore, only particle dispersions at *pH* 3 to *pH* 7 (*pH* 6 for NP E) were subjected to further analysis.

**Figure 4:**
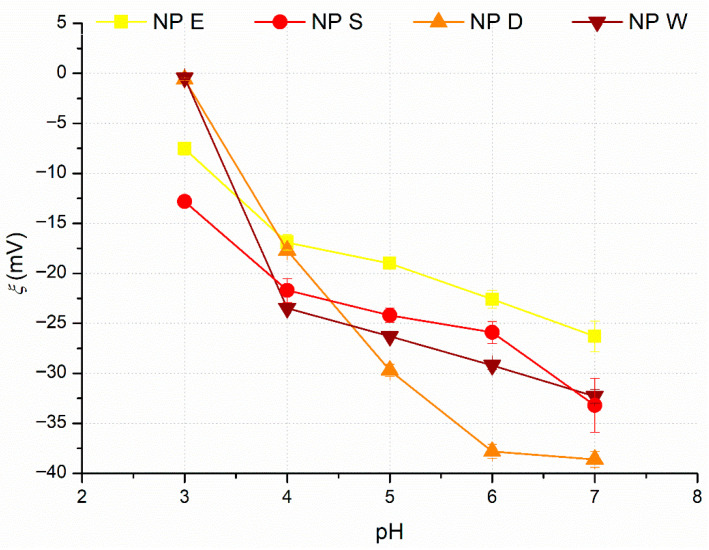
Influence of *pH* on the zeta potential, *ζ*, of the cutin dispersions NP E, NP S, NP D, and NP W.

Zeta potential, *ζ*, of the dispersions NP E, NP S, NP D, and NP W as a function of *pH* is illustrated in Figure 4.

Particles of all four cutin dispersions (NP E, NP S, NP D, and NP W) bear negative charge, which becomes more negative as *pH* increases from *pH* 3 to *pH* 9. Cutin particles from the NP D and NP W dispersions at *pH* = 3 have the least negative charge ≈ 0 mV, while the most charged nanoparticles are nanoparticles from NP D dispersion at *pH* 6 and *pH* 7 with ζ of −37.8 ± 0.7 mV and −38.6 ± 0.8 mV, respectively. The negative charge can be attributed to the ionization of carboxylic groups of C16–C18 interesterified fatty acid derivatives, which make up the main component of cutin [13,15,17,21]. The obtained results are similar to the results reported by Manrich et al. (2017) [17], who measured the influence of *pH* on *ζ* of cutin nanoparticles prepared by the top-down approach, apart from particles at *pH* 3, which had positive zeta potential.

**Figure 5:**
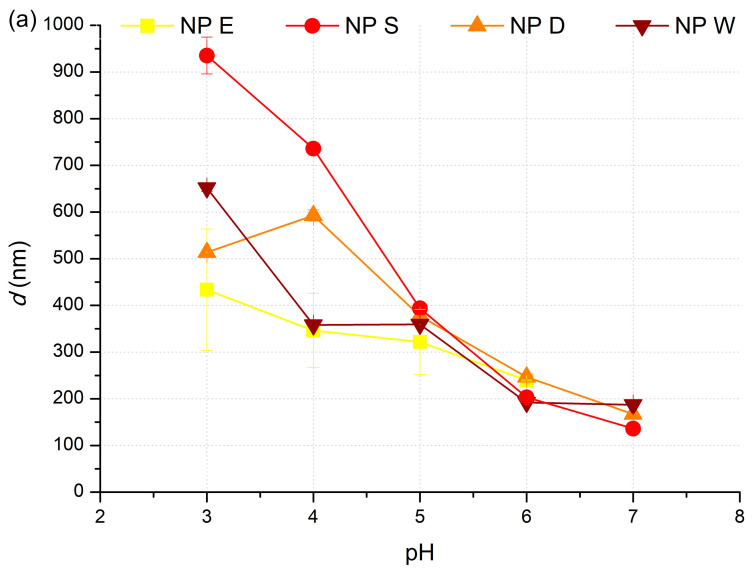

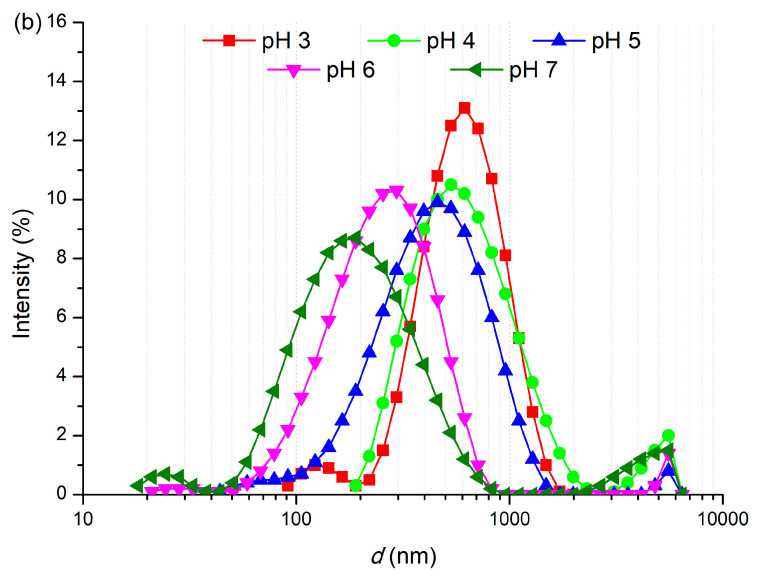
Influence of *pH* on (**a**) mean particle diameter, *d*, of the particles in dispersions NP E, NP S, NP D, NP W and on (**b**) particle size distribution of NP D. *c_S_* = 2%.

The influence of *pH* on mean particle diameter, *d*, of cutin particles in dispersions NP E, NP S, NP D, and NP W, and an exemplary particle size distribution of NP D, is presented in Figure 5a,b, respectively.

As Figure 5a shows, the mean particle diameter of all four types of cutin particles is under 1 µm and it decreases upon *pH* increase from 3 to 9. Cutin particle preparation route and type of cutin isolate (I_d_, I_s_, and I_w_) used for the preparation of cutin dispersion resulted in significant difference in *d* of cutin particles among NP E, NP S, NP D, and NP W dispersions at *pH* 3 and *pH* 4. Therefore, at *pH* ≤ 4, *d* of cutin particles in different dispersions (NP E, NP S, NP D, and NP W) varies by a couple of hundred of nanometers at one *pH*, being the lowest in dispersion NP E and the largest in NP S. On the other hand, at *pH* ≥ 5, the influence of dispersion type on *d* of cutin particles is minimized so *d* of cutin particles is in the range of 320–390 nm at *pH* 5, in the range of 190–250 nm at *pH* 6, and in the range of 140–190 nm at *pH* 7, regardless of the nanoparticle type. The decrease in *d* upon the increase in *pH* is the result of the increase in zeta potential (Figure 4), i.e., increase in electrostatic repulsion, which hinders the interactions between polymer chains or intra-chain interaction and the formation of larger aggregates during precipitation [29,30]. Nevertheless, *d* of cutin nanoparticles in dispersions NP E, NP D, NP S, and NP W has shown to be much smaller than *d* of cutin particles prepared by Manrich et al. (2017) [17], whose top-down technique resulted in cutin particles with *d* of ≈6000 nm at *pH* = 3, with a trend of decrease as *pH* increases. Finally, *d* becomes smaller than 1000 nm at *pH* ≥ 6.

Size distribution of cutin particles at *pH* 3–7 is shown on an example of NP D (Figure 5b). Cutin particles in dispersions NP E, NP S, NP D, and NP W have monomodal size distribution with a “shoulder” at *d* lower than 100 nm, regardless of *pH*. Increase in *pH* from *pH* 3 to *pH* 7 resulted in a shift of the main pick towards larger particle sizes.

### 3.3. Cutin Particle Recovery

**Figure 6:**
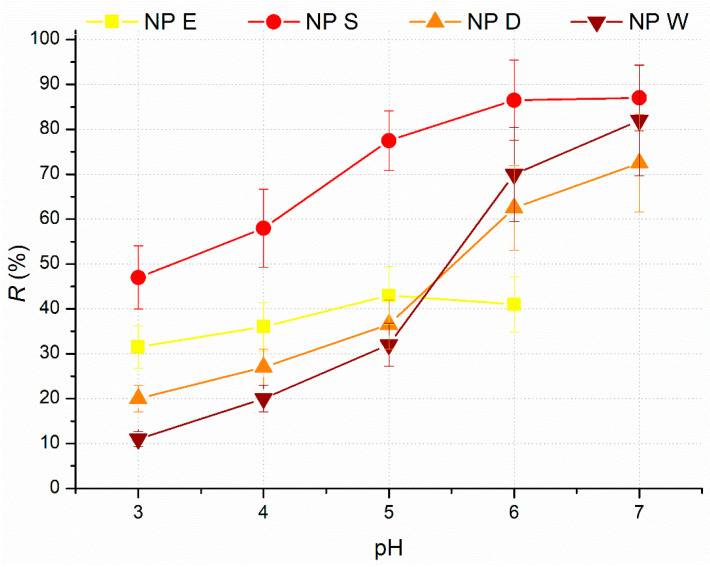
Influence of *pH* on recovery, R, of cutin particles in NP E, NP D, NP S, and NP W. *c_s_* = 2%.

The efficacy of different routes to prepare cutin nanoparticles was evaluated by determining a cutin particle recovery, *R*. The influence of *pH* on *R* of cutin particles in NP E, NP D, NP S, and NP W is presented in Figure 6.

Recovery of cutin particles in all four dispersions increases as *pH* increases from 3 to 7. The highest *R* among dispersions NP E, NP S, NP D, and NP W was obtained for NP S at each *pH* investigated. *pH* has considerable influence on *R* in all four dispersions with differences between *pH* 3 and *pH* 7 for different cutin dispersions following the order ≈10%, ≈40%, ≈50%, and ≈70% for dispersions NP E, NP S, NP D, and NP W, respectively. Such differences in *R*, depending on the method used for nanoparticle preparation, were reported in the production of starch nanoparticles, where *R* ranges from 15% to 100% [11].

### 3.4. Influence of the Cutin Solution Concentration on Cutin Particle Size

**Figure 7:**
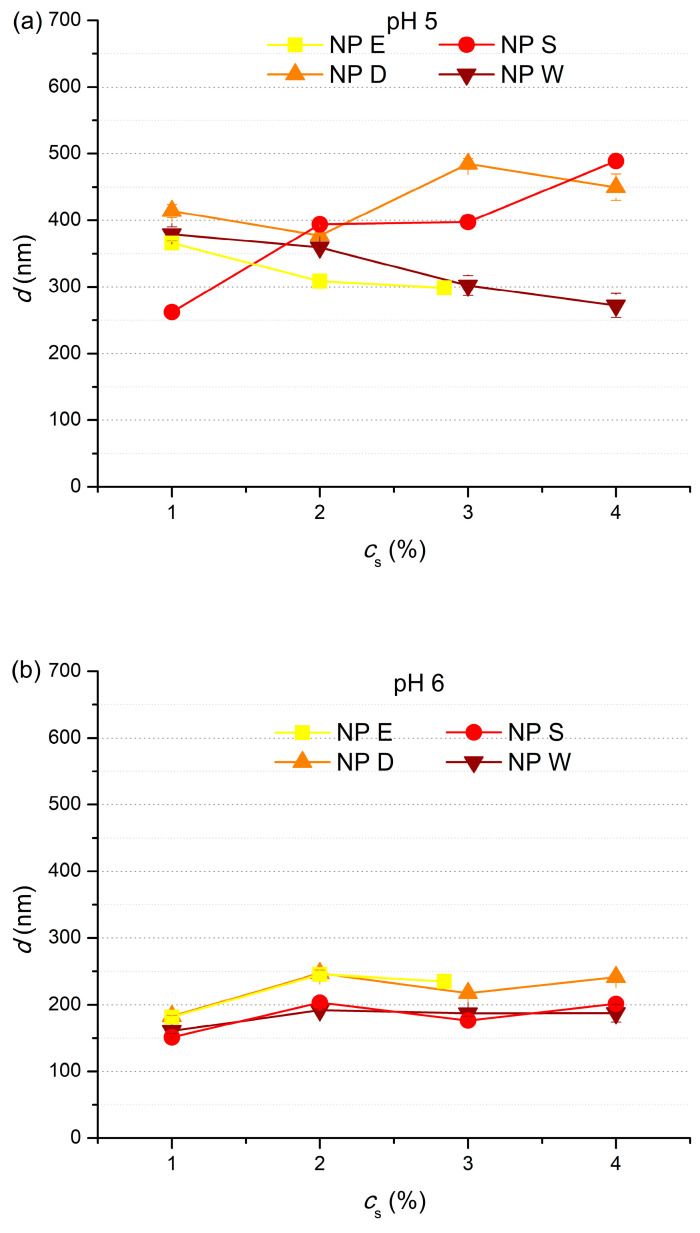

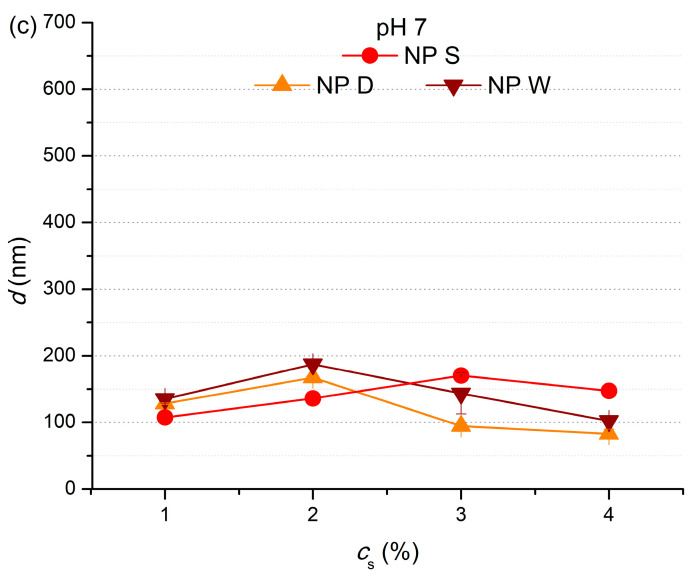
Influence of cutin solution concentration, *c_s_*, on cutin particle size in NP E, NP S, NP D, and NP W at (**a**) *pH* 5, (**b**) *pH* 6, and (**c**) *pH* 7.

Cutin solutions of different concentrations were precipitated by decreasing *pH* of the solutions in order to obtain cutin nanoparticles. The influence of cutin solution concentration (*c_s_* = 1–4%) on cutin particle size for different dispersions (NP E, NP S, NP D, and NP W) and different final *pH* (5, 6, 7) was investigated (Figure 7).

Cutin solution concentration has the strongest influence on cutin particle size at *pH* 5 (Figure 7a), regardless of cutin dispersion type. At *pH* = 5, particle size in cutin dispersions NP S and NP D increases upon the *c_s_* increase from 1% to 4%, while *d* of particles in dispersions NP E and NP W decreases. Such results can be attributed to different stages of the same effects. Namely, increase in particle size is attributed to increased viscosity of cutin solutions when concentration is increased, which flavors the formation of larger particles during the precipitation [3,5]. On the other hand, the decrease in the cutin particle size can be explained by the progression of the effect of increased viscosity to the point when formed particles are large enough to be removed from the cutin dispersion during the final filtration, which is indicated by lower *R* at *pH* 5 in comparison to the *R* at higher *pH* (Figure 6).

Figure 7b shows that at *pH* 6, the increase in *c_s_* brought about a slight increase in cutin particle size in all four dispersions, while at *pH* 7 (Figure 7c), *d* of cutin particles in NP S, NP D, and NP W ranged from 80 nm to 190 nm, regardless of *c_s_*. The decrease in influence of *c_s_* as *pH* increases from 5 to 7 comes from the increased charge of cutin particles upon *pH* increase (Figure 4); therefore, at *pH* ≥ 6, cutin particle charge becomes strong enough to minimize the effect of the increased viscosity upon the increase in *c_s_*.

### 3.5. Influence of Cutin Dispersion Storage Time on Particle Size

The stability of NP E, NP S, NP D, and NP W was monitored during 14 days of storage at room temperature.

**Figure 8:**
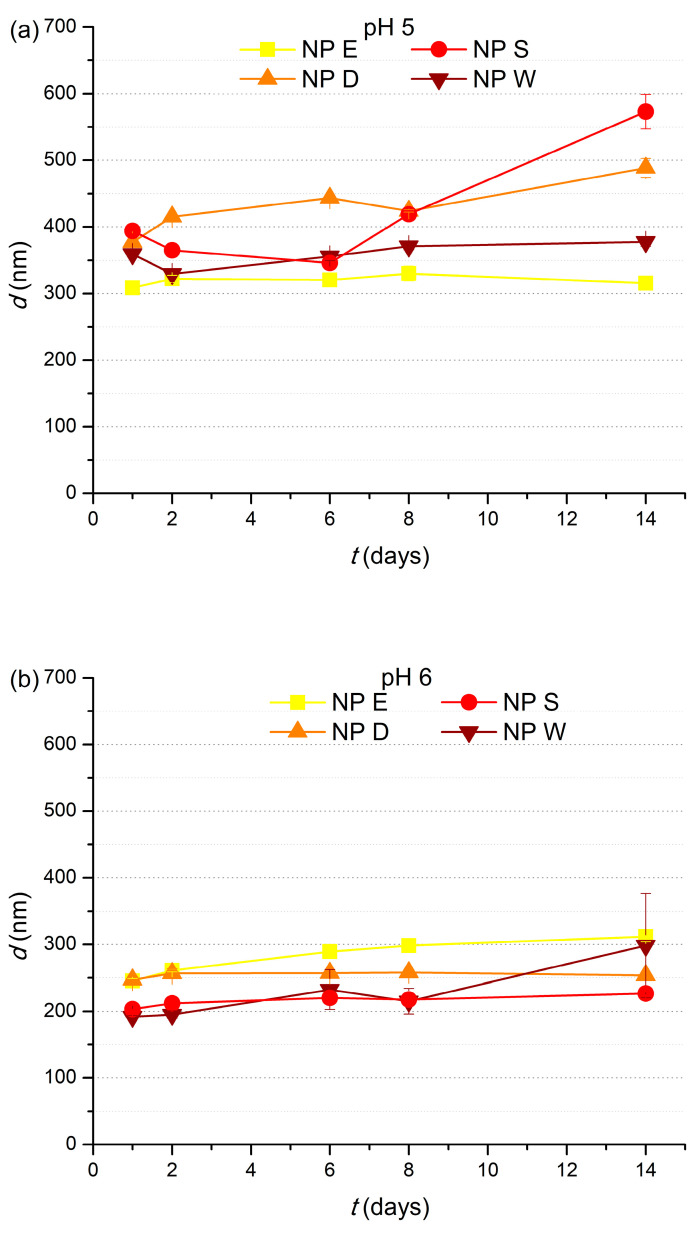

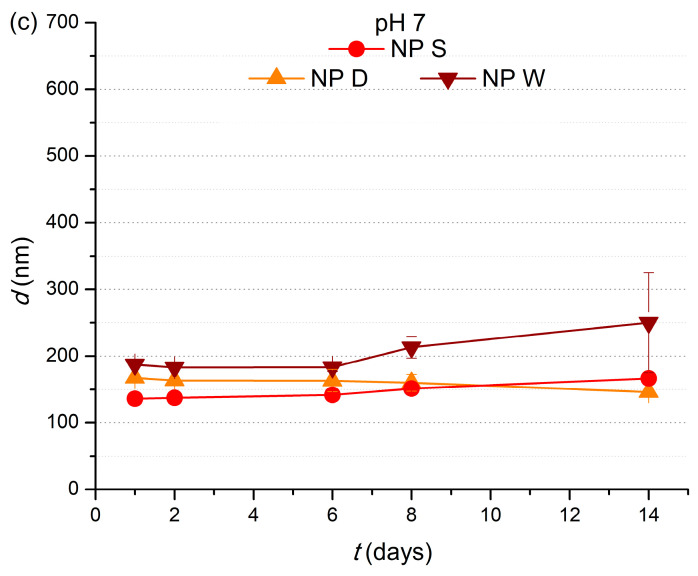
Influence of storage time on *d* of four cutin dispersions, NP E, NP S, NP D, and NP W, at (**a**) *pH* 5, (**b**) *pH* 6, and (**c**) *pH* 7. *c_s_* = 2%.

The influence of cutin dispersion storage time on particle size at different final *pH* of 5, 6, and 7 is presented in Figure 8a–c, respectively.

At *pH* 5, *d* of NP E and NP W varied only slightly during the 14 days of storage; however, the *d* of NP S and NP D increased significantly, from 393.8 ± 4.14 nm to 573.0 ± 26 nm and from 377.3 ± 1.8 nm to 488.5 ± 14.12 nm, respectively. Cutin particle size in dispersions NP E, NP S, NP D, and NP W at *pH* 6 (Figure 8b) slightly increased, while at *pH* 7 (Figure 8c), storage time had negligible influence on *d* of cutin particles in dispersions NP S and NP D and only slightly increased *d* of cutin particles in dispersion NP W. The colloidal stability of cutin dispersions and the decrease of the influence of storage time on *d* of NP E, NP S, NP D, and NP W as *pH* increases from 5 to 7 can be attributed to the relatively high zeta potential of NP E, NP S, NP D, and NP W that increases as *pH* increases (Figure 4), providing enough electrostatic repulsion among nanoparticles to prevent aggregation [29].

## 4. Conclusions

Cutin nanoparticles were formed by pH-dependent precipitation, at *pH* ≤ 7, either from cutin extract obtained directly from tomato peels—nanoparticle dispersion NP E, or from cutin solution of three different cutin isolates: standard cutin isolate—nanoparticle dispersion NP S, dialyzed cutin isolate—nanoparticle dispersion NP D, and washed cutin isolate—nanoparticle dispersion NP W. Recovery of cutin nanoparticles in the four dispersions was found to increase from *pH* 3 to *pH* 7, with the dispersion NP S having the highest recovery at all *pH*s investigated. Cutin nanoparticles were found to bear negative charge which increase with *pH* increase, reaching the highest value at the *pH* of precipitation start, *pH* = 7, regardless of the cutin nanoparticle dispersion type. In accordance with the cutin nanoparticle zeta potential, particle size decreases as *pH* increases. Mean cutin nanoparticle diameter, d, in dispersions NP E, NP S, NP D, and NP W is significantly affected by *pH* only at *pH* ≤ 4, while the influence of *pH* at *pH* ≥ 5 is mitigated. Influence of the cutin solution concentration, c_s_ (1–4%) on d of NP E, NP S, NP D, and NP W was investigated at *pH* 5, 6, and 7. The obtained results show that at *pH* 5, increase in c_s_ resulted in increase in d of NP S and NP D while d of NP E and NP W decreased. This is attributed to the effect of increased viscosity that favors the formation of larger particles, which in dispersions NP E and NP W become large enough to be removed by final dispersion filtration. At *pH* 6 and *pH* 7, the effect of the increase in c_s_ was less emphasized due to the increased charge of cutin nanoparticles, which improved the electrostatic stabilization of the cutin nanoparticles. The influence of the storage time of cutin nanoparticle dispersions at *pH* 5, 6, and 7 was monitored for 14 days at room temperature. The highest influence of storage time on cutin nanoparticle size was determined at *pH* 5, while as *pH* increases to 7 the influence of storage time on d decreases. Once again, the mitigated effect of storage time on cutin nanoparticle size on increase in *pH* is a result of increased cutin nanoparticle charge at higher *pH*. The results obtained show that tomato peels can be successfully processed into stable cutin nanoparticle dispersions, which are ready for further application as coatings, emulsion stabilizers, or nanoencapsulation; however, this remains a subject for future investigation.

## Data Availability

The raw data is available from the corresponding author by request.

## Abbreviations

The following abbreviations are used in this manuscript:

NP E	nanoparticle dispersion obtained from cutin extract
NP D	nanoparticle dispersion obtained from dialyzed cutin isolate
NP S	nanoparticle dispersion obtained from standard cutin isolate
NP W	nanoparticle dispersion obtained from washed cutin isolate
Is	standard cutin isolate
Iw	washed cutin isolate
Id	dialyzed cutin isolate
R	recovery
cs	cutin solution concentration
